# Symptom networks in major depressive disorder and treatment response: special focus on TRD

**DOI:** 10.1192/j.eurpsy.2025.2454

**Published:** 2025-05-27

**Authors:** Alexander Kautzky, Lucie Bartova, Markus Dold, Daniel Souery, Stuart Montgomery, Joseph Zohar, Julien Mendlewicz, Chiara Fabbri, Alessandro Serretti, Evgenii Tretiakov, Dan Rujescu, Tibor Harkany, Siegfried Kasper

**Affiliations:** 1 Department of Psychiatry and Psychotherapy, Clinical Division of General Psychiatry, Medical University of Vienna, Vienna, Austria; 2 Department of Clinical Neurosciences, Division of Insurance Medicine, Stockholm, Sweden; 3 Comprehensive Center for Clinical Neurosciences and Mental Health, Medical University of Vienna, Vienna, Austria; 4 Laboratoire de Psychologie Medicale, Université Libre de Bruxelles and Psy Pluriel Centre Europèen de Psychologie Medicale, Brussels, Belgium; 5 Imperial College, University of London, London, UK; 6 Psychiatric Division, Chaim Sheba Medical Center, Tel Hashomer, Israel; 7 School of Medicine, Free University of Brussels, Brussels, Belgium; 8 Department of Biomedical and Neuromotor Sciences, University of Bologna, Bologna, Italy; 9 Department of Medicine and Surgery, Kore University of Enna, Enna, Italy; 10 Department of Molecular Neurosciences, Center for Brain Research, Medical University of Vienna, Vienna, Austria

**Keywords:** major depressive disorder, treatment-resistant depression, antidepressants, network analysis, symptom networks

## Abstract

**Background:**

Heterogeneous symptoms in major depression contribute to unsuccessful antidepressant treatment, termed treatment-resistant depression (TRD). Psychometric network modeling conceptualizes depression as interplay of symptoms with potential benefits for treatment; however, a knowledge gap exists regarding networks in TRD.

**Methods:**

Symptoms from 1,385 depressed patients, assessed by the Montgomery-Åsberg-depression rating scale (MADRS) as part of the “TRD-III” cohort of the multinational research consortium “Group for the Studies of Resistant Depression,” were used for Gaussian graphical network modeling. Networks were estimated for two timepoints, pretreatment and posttreatment, after the establishment of outcomes response, non-response, and TRD. Applying the network-comparison test, edge weights, and symptom centrality was assessed by bootstrapping. Applying the network-comparison test, outcome groups were compared cross-sectionally and longitudinally regarding the networks’ global strength, invariance, and centrality.

**Results:**

Pretreatment networks did not differ in global strength, but outcome groups showed distinct symptom connections. For both response and TRD, global strength was reduced posttreatment, leading to significant differences between each pair of networks posttreatment. Sadness, lassitude, inability-to-feel, and pessimistic thoughts ranked most centrally in unfavorable outcomes, while reduced-appetite and suicidal thoughts were more densely connected in response. Connections between central symptoms increased in strength following unsuccessful treatment, particularly regarding links involving pessimistic thoughts in TRD.

**Conclusion:**

Treatment reduced global network strength across outcome groups. However, distinct symptom networks were found in patients showing response to treatment, non-response, and TRD. More easily targetable symptoms such as reduced-appetite were central to networks in patients with response, while pessimistic thoughts may be a key symptom upholding disease burden in TRD.

## Introduction

Despite major depressive disorder (MDD) being highly prevalent [1–3], the neurobiological underpinnings remain under investigation and treatment options are still suboptimal [4, 5]. Effective treatment options include antidepressants (AD) targeting monoamine reuptake and receptor binding [6], as well as glutamatergic signaling and neuroplasticity [7]. Nevertheless, many patients do not show sufficient symptom relief even after multiple treatment trials and develop treatment-resistant depression (TRD) [8]. TRD has been extensively studied and linked to various genetic [9, 10], environmental [11], and clinical characteristics [12]. The framework of the research consortium “European group for the study of resistant depression” (GSRD), consisting of consecutive waves of patients with severe MDD whose treatment was outcome assessed across multiple trials, has contributed risk factors for TRD [13, 14]. However, there are still no validated clinical tools for early risk assessment of TRD and, more importantly, optimal treatment choices to prevent TRD. Heterogeneity in the neurobiological and environmental pathways leading to depression have been identified as obstacles in finding consistent diagnostic and prognostic markers in depression. A related important factor is the lack of a representation of the plethora of depressive symptoms and their respective contributions to the variability in treatment outcomes [15].

Targeting depression on the symptom level and finding patterns of co-expression has gained interest in recent years [16]. Symptom networks highlight influential symptoms that are central to the clinical presentation of MDD [17], distinct to a subgroup of patients, or specific in response to treatment [2]. Results were shown to replicate and may bring more precision to treatment choices [18]. However, studies comparing network characteristics before and after antidepressant treatment are still scarce [19–21] and implications of symptom networks remain under discussion [22, 23]. The concept of TRD itself was recently challenged as empty [24], however, symptom patterns specific to TRD that may inform targeted treatment have not been analyzed yet. Thus, we aimed to compare symptom networks in treatment response, non-response, and TRD in patients with MDD and to identify symptoms central to specific outcomes.

## Methods

### Sample

The TRD-III cohort of the GSRD project was used for network generation [13], including a total of 1,410 patients with MDD recruited 2011–2016 as in- or outpatients in 15 community and university hospitals across Europe and Israel. Diagnoses were established with the Mini International Neuropsychiatric Interview (MINI) and MDD was required to be the primary diagnosis [25]. Depression severity was assessed using the Montgomery-Åsberg-depression rating scale (MADRS) for two timepoints [26]: pretreatment and when response, non-response, or TRD were established. The pretreatment MADRS score was assessed retrospectively based on medical records and patients´ recollection of symptom severity at the time of treatment initiation. At least moderate depression severity according to a pretreatment MADRS score > 21 was required.

The European staging system for treatment outcome was used [27]:

Treatment response was defined by a decline ≥50% in MADRS score after an adequate antidepressant treatment trial compared to pretreatment scores assessed retrospectively. If response was not reached after a single AD trial of adequate length and dosage, outcome was labeled non-response. If response was not achieved after two or more antidepressant trials were administered, outcome was labeled TRD. Detailed descriptions of the TRD-III sample regarding clinical characteristics and outcomes have been published previously [13].

The authors assert that all procedures contributing to this work comply with the ethical standards of the relevant national and institutional committees on human experimentation and with the Helsinki Declaration of 1975, as revised in 2013. All procedures involving human subjects/patients were approved by the Ethics Committee of the Medical University of Vienna (EK 1133/11).

### Network estimation

Symptom networks were estimated with the “bootnet” package for the statistical program “R” (version 4.3.0) [3]. Following published recommendations [28], matrices of Spearman correlations between each symptom pair were used to estimate sparse Gaussian graphical models. Nodes correspond to depressive symptoms according to the 10 MADRS items and edges to their partial correlation. The mean score of items 1 and 2, both addressing sadness, was used, totaling 9 nodes and a maximum of 36 interconnecting edges. The most parsimonious model, i.e., the model with optimal performance while minimizing the number of edges, was determined by optimization of the extended Bayesian information criterium by graphic L1 regularization (EBIC-lasso). The default parameter of λ = 0.5 was applied. Network stability and accuracy of edge weights were assessed by bootstrapping following published recommendations for “bootnet” [3]. Detailed information on bootstrapping procedures and assessment of Berkson’s bias can be found in Supplementary Section [29].

Next, strength was assessed for each node of a network by summing up the weights of all connected edges. Thus, node strength indicates the centrality of a specific symptom regarding all other symptoms of a network.

Networks were then visualized with the “qgraph” package [30]. Thereby, all edges estimated by EBIC-lasso were visualized. Edge weights, ranging between 0 and 1, were labeled for each stable edge according to bootstrapping. In addition, strength indices for each node of each network were visualized as line plots.

### Network comparison

To assess differences in network structures between each pair of networks, the R-package “NetworkComparisonTest” (NCT) was used to compute the statistics S (difference in global network strength) and M (network invariance assessed by maximum difference in edge weights) [31]. We compared between-subjects networks estimated for each treatment outcome group, respectively pre- and posttreatment, as well as within-subjects networks for symptoms reported pre- and posttreatment within each outcome group. The latter comparisons were computed with the experimental “paired” option of NCT. Furthermore, for each significant comparison of two networks according to *S* and *M* statistics, differences in individual edges (whenever *M* < 0.05) and node strength (whenever *S* < 0.05) were tested. Edges were compared only when they were stable according to bootstrapping in both networks. For each NCT result, uncorrected *p*-values are reported with a significance level < 0.05.

## Results

MADRS ratings were fully available for 1,385 patients (66.8% female, mean age 51.7 ± 14.1 years), including 326 responders (pretreatment MADRS 32.6 ± 8.0), 489 non-responders (pretreatment MADRS 33.2 ± 8.1), and 570 patients with TRD (pretreatment MADRS 35.8 ± 6.7). Details on age, sex, and presentation of depressive symptoms both pre- and posttreatment are provided in Table 1. The distribution of MADRS sum scores and individual items, pre- and posttreatment, is displayed in Supplementary Figures 1–3. For a distribution of severe symptoms (MADRS item score > 3), for each outcome and pre- and posttreatment, see Supplementary Table 1.

**Table 1.** Sex, age, and symptom presentation for the three treatment outcomes, shown both pre- and posttreatment

Group	Response (*n* = 326)		TRD (*n* = 570)		Non-response (*n* = 489)	
Timepoint	Pre	Post	Pre	Post	Pre	Post
Sex						
Female	213 (65.3%)		370 (64.9%)		341 (69.7%)	
Male	113 (34.7%)		200 (35.1%)		148 (30.3%)	
Age						
Mean (SD)	51.2 (15.9)		52.1 (13.6)		51.5 (13.2)	
Median (range)	51.0 [21.0, 91.0]		52.0 [21.0, 93.0]		52.0 [21.0, 91.0]	
Sadness (MADRS #1 + 2)						
Mean	4.0 (1.0)	1.1 (0.9)	4.4 (0.8)	4.0 (0.9)	4.1 (0.9)	3.5 (1.0)
Median	4.0 [0.5–6.0]	1.0 [0.0–4.0]	4.5 [1.0–6.0]	4.0 [1.0–6.0]	4.0 [0.0–6.0]	3.5 [0.0–6.0]
Inner tension (MADRS #3)						
Mean	3.4 (1.3)	1.1 (1.0)	3.6 (1.1)	3.1 (1.1)	3.5 (1.2)	2.8 (1.1)
Median	4.0 [0.0–6.0]	1.0 [0.0–5.0]	4.0 [0.0–6.0]	3.0 [0.0–6.0]	4.0 [0.0–6.0]	3.0 [0.0–6.0]
Reduced sleep (MADRS #4)						
Mean	3.4 (1.5)	0.8 (1.0)	3.7 (1.4)	3.1 (1.5)	3.5 (1.4)	3.0 (1.5)
Median	4.0 [0.0–6.0]	0 [0.0–4.0]	4.0 [0.0–6.0]	4.0 [0.0–6.0]	4.0 [0.0–6.0]	3.0 [0.0–6.0]
Reduced appetite (MADRS #5)						
Mean	2.3 (1.8)	0.3 (0.8)	2.3 (1.7)	1.8 (1.5)	2.0 (1.7)	1.63 (1.5)
Median	2.0 [0.0–6.0]	0 [0.0–6.0]	2.0 [0.0–6.0]	2.0 [0.0–6.0]	2.0 [0.0–6.0]	1.0 [0.0–6.0]
Concentration difficulties (MADRS #6)						
Mean	3.5 (1.3)	1.1 (1.1)	3.7 (1.0)	3.3 (1.0)	3.5 (1.2)	3.1 (1.1)
Median	3.0 [0.0–6.0]	1.0 [0.0–4.0]	4.0 [0.0–6.0]	3.0 [0.0–6.0]	4.0 [0.0–6.0]	3.0 [0.0–6.0]
Lassitude (MADRS #7)						
Mean	3.7 (1.2)	1.0 (0.9)	4.1 (0.9)	3.7 (1.0)	3.8 (1.2)	3.2 (1.1)
Median	4.0 [0.0–6.0]	1.0 [0.0–4.0]	4.0 [0.0–6.0]	4.0 [0.0–6.0]	4.0 [0.0–6.0]	3.0 [0.0–6.0]
Inability to feel (MADRS #8)						
Mean	3.4 (1.2)	0.7 (1.0)	3.8 (1.1)	3.3 (1.1)	3.5 (1.2)	3.0 (1.2)
Median	3.0 [0.0–6.0]	0 [0.0–4.0]	4.0 [0.0–6.0]	3.0 [0.0–6.0]	4.0 [0.0–6.0]	3.0 [0.0–6.0]
Pessimism (MADRS #9)						
Mean	3.1 (1.3)	1.0 (1.0)	3.7 (1.2)	3.3 (1.1)	3.3 (1.3)	2.9 (1.2)
Median	3.0 [0.0–6.0]	1.0 [0.0–4.0]	4.0 [0.0–6.0]	4.0 [0.0–6.0]	3.0 [0.0–6.0]	3.0 [0.0–6.0]
Suicidal thoughts (MADRS #10)						
Mean	1.8 (1.8)	0.2 (0.5)	2.1 (1.7)	1.7 (1.5)	1.8 (1.7)	1.4 (1.4)
Median	1.0 [0.0–6.0]	0 [0.0–4.0]	2.0 [0.0–6.0]	1.0 [0.0–6.0]	1.0 [0.0–6.0]	1.0 [0.0–6.0]

*Note*: Means are shown with standard deviations; medians, with range.

### Network structure pretreatment

**Figure 1.**
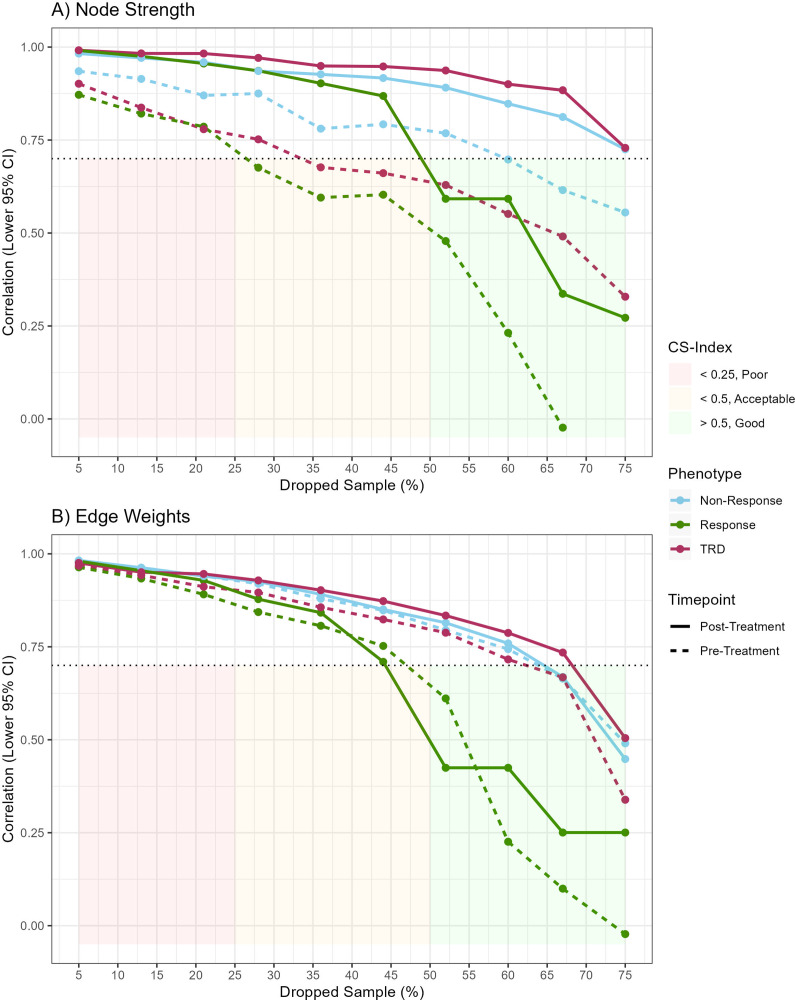
*Case-dropping subset bootstrap* results. On the x-axis the proportion of dropped cases is shown, while the y-axis indicates the lower 95% confidence interval (CI) of the correlation coefficient between the 1,000 bootstrapped samples (termed *correlation stability* (CS) coefficient), respectively for (A) node strengths and (B) edge weights. The cutoff of 0.7 is marked by a dotted line and colored areas under this cutoff indicate poor, acceptable, and good stability. Colored lines indicate the achieved correlation coefficients for each network, for pre- and posttreatment and the phenotypes of treatment-resistant depression (TRD), non-response, and response.

**Figure 2.**
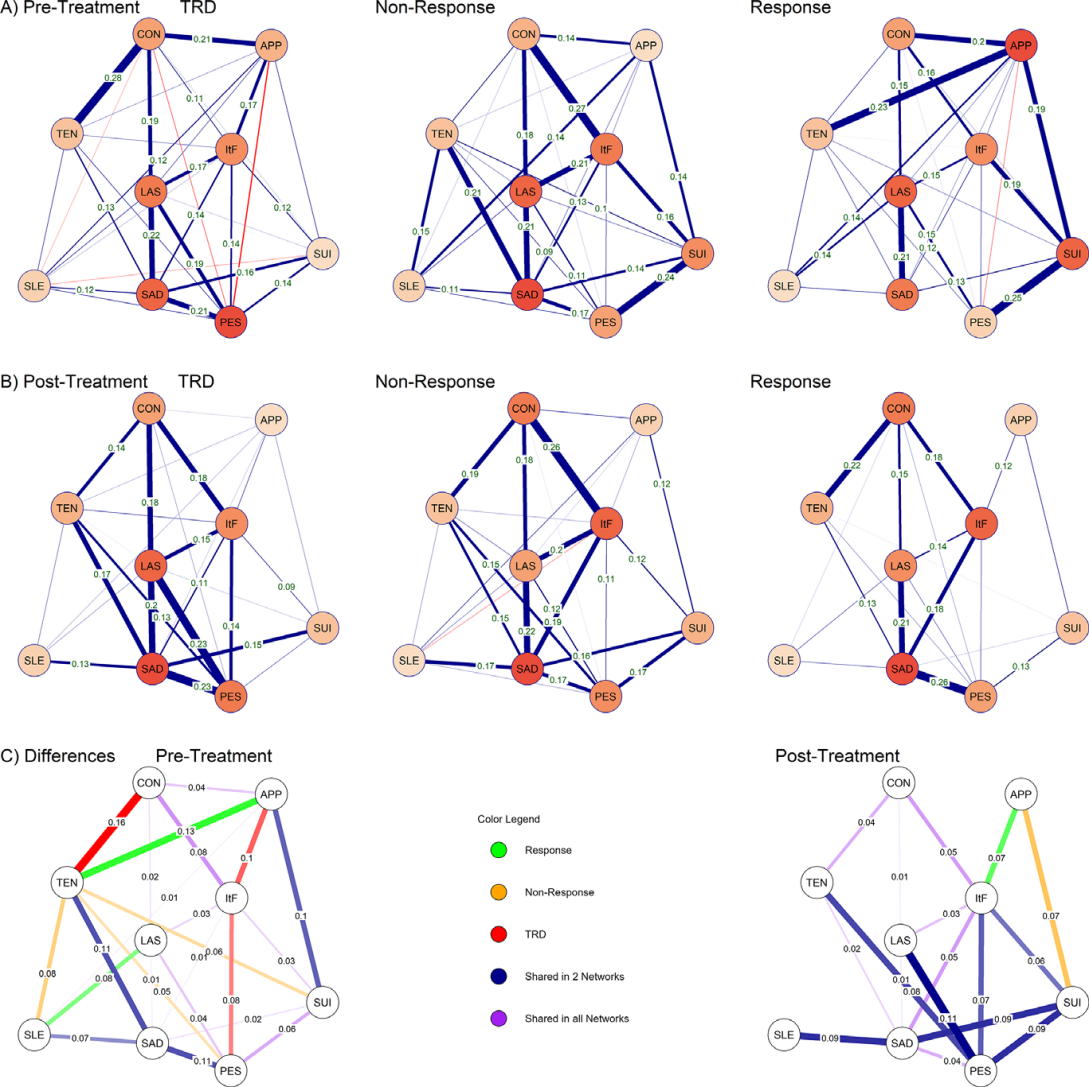
Gaussian graphical models estimated for patients with TRD, non-, and response to treatment for two timepoints, (A) before initiation of antidepressant treatment, and (B) after treatment outcome was determined. Positive partial correlations, i.e., co-expression of severity of two symptoms, are portrayed in blue color while negative partial correlations, i.e., high load of one symptom occurring with low severity of another, are portrayed in red color. In panel (C), standard deviations from mean edge weights of the three networks estimated for pre- and posttreatment are displayed. Colors correspond to presence of edges in specific or multiple outcome phenotypes. In all networks, edge weights are only displayed when stable according to bootstrapping. Nodes are colored dark to light by declining node strength, i.e., the sum of edge weights connecting each node. Abbreviations: APP, reduced-appetite; CON, concentration difficulties; ItF, inability-to-feel; LAS, lassitude; PES, pessimistic thoughts; SAD, sadness; SLE, reduced-sleep; SUI, suicidal thoughts; TEN, tension; TRD, treatment-resistant depression.

Stability of edge weights was good for networks estimated, respectively for patients with TRD and non-responders, and acceptable in the group of treatment responders (Figure 1). Stability of node strength was good in non-responders and acceptable in patients with TRD and responders. Networks estimated on symptoms pretreatment are depicted in Figure 2A. No significant differences were detected by NCT regarding global strength, ranging from 3.5 in responders to 3.9 in TRD patients (all *p* > 0.05). Network invariance was not significantly different between networks in TRD and non-responders, but it was different between responders and TRD (*M* = 0.21, *p* = 0.043), and between responders and non-responders (*M* = 0.21, *p* = 0.037). No significant differences were observed between individual stable edges.

Stable edges between lassitude sadness, concentration difficulties and inability-to-feel, as well as between pessimistic thoughts and suicidal thoughts, were shared between all baseline networks (Figure 2B). Within all three networks, the strongest edges as well as nodes were not significantly different from each other, but only compared to the lower-ranking edges and nodes (Supplementary Figure 4).

**Figure 3.**
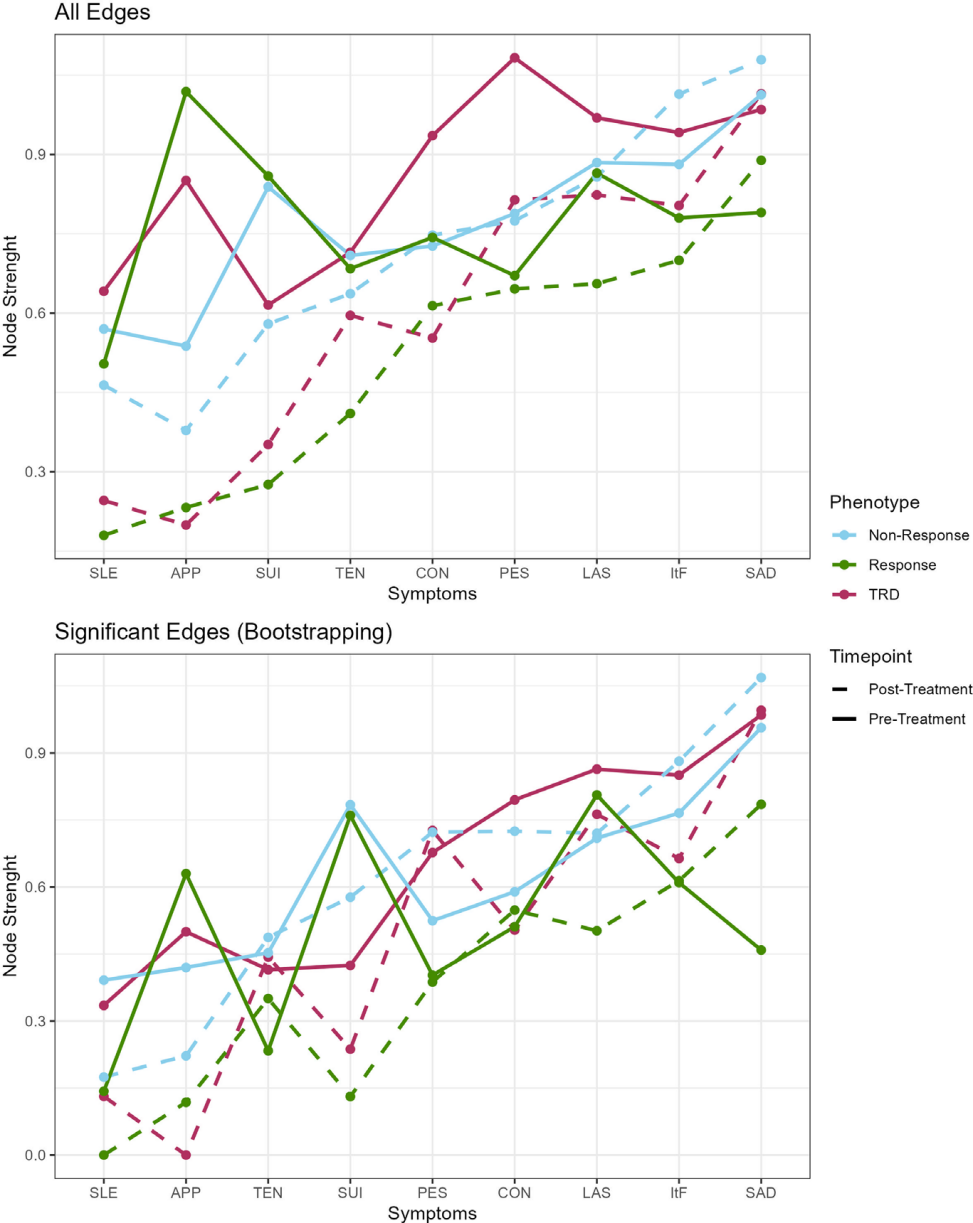
Symptom centrality assessed by node strength, that is, the sum of edge weights connecting a specific symptom directly to other symptoms. Results are colored by treatment outcomes TRD, non-response, and response. Panel (A) shows strength rankings for edges, while panel (B) shows rankings when restricting strength to stable edges with non-zero confidence intervals according to bootstrapping. APP, reduced-appetite; CON, concentration difficulties; ItF, inability-to-feel; LAS, lassitude; PES, pessimistic thoughts; SAD, sadness; SLE, reduced-sleep; SUI, suicidal thoughts; TEN, tension; TRD, treatment-resistant depression.

In patients with TRD, a network with 32 edges (18 stable) was estimated. The strongest edge was observed between tension and concentration difficulties, which was unique to TRD. Regarding node strength, pessimistic thoughts ranked before sadness, lassitude, inability-to-feel, and concentration difficulties (Figure 3A). When considering only stable edges (Figure 3B), lower node strength was observed for pessimism.

In patients with treatment non-response 30 edges (17 stable) were estimated, with the strongest edge between concentration difficulties and inability-to-feel, which was significant also in the groups of TRD and responders. Unique edges linked tension to sleep, pessimistic thoughts, and suicidal thoughts. Regarding node strength, sadness ranked before lassitude, inability-to-feel, suicidal thoughts, and pessimistic thoughts. When considering only stable edges, concentration difficulties ranked before pessimistic thoughts.

In patients with treatment response, 30 edges (13 stable) were estimated. The strongest edge linked reduced-appetite to tension, which was unique to the response network. A second unique edge was found between reduced-sleep and lassitude. Highest node strength was found for reduced-appetite, lassitude, suicidal thoughts, concentration difficulties, and inability-to-feel. A lower ranking was observed for reduced-appetite when considering only stable edges.

### Network structure posttreatment

Stability of both edge weights and node strength was good for networks estimated for patients with TRD and non-responders, and acceptable in the group of treatment responders (Figure 1). Networks are depicted in Figure 2C. Networks differed in global strength, ranging from 2.3 in responders to 3.2 in non-responders (all *p* < 0.05). For individual node strengths differing between networks, please see Table 2, panel B. Structure was not different between any networks (all *p* > 0.05).

**Table 2.** Results of the network comparison tests, respectively for cross-sectional comparisons between treatment outcomes and longitudinal comparisons within each treatment outcome

Timepoint	Comparison	Network invariance		Global strength	
		*M*	*p*	*S*	*p*
**(A) Network comparisons**					
**Pretreatment**	TRD vs. non-resp.	0.19	n.s.	0.39	n.s.
	TRD vs. resp.	0.21	0.043	0.41	n.s.
	Non-resp. vs. resp.	0.21	0.037	0.02	n.s.
**Posttreatment**	TRD vs. non-resp.	0.12	n.s.	0.57	0.042
	TRD vs resp.	0.15	n.s.	0.4	0.011
	Non-resp. vs. resp.	0.14	n.s.	0.96	<0.001
**Longitudinal**	TRD	0.2	<0.001	1.17	<0.001
	Non-response	0.1	n.s.	0.21	n.s.
	Response	0.26	0.026	1.16	<0.001
**Timepoint**	**Comparison**	**Nodes**	**p**	**Edges**	**p**
**(B) Post hoc**					
**Posttreatment**	TRD vs. non-resp.	SUI	0.033	Not tested	
	TRD vs. resp.	TEN	0.034		
	Non-resp. vs. resp.	TEN	0.015		
		SLE	0.011		
		LAS	0.044		
		ItF	0.001		
		SUI	<0.001		
**Longitudinal**	TRD	SLE	0.005	TEN-CON	0.01
		APP	<0.001		
		CON	0.003		
		PES	0.031		
		SUI	0.008		
	Response	TEN	0.025	n.s.	
		SLE	0.003		
		APP	<0.001		
		SUI	<0.001		

*Note*: (A) Global tests included network invariance, that is, whether network structures are different, as well as network strength, that is, whether node strengths are different. (B) In case of significant global results, individual edges or nodes were tested.

n.s. not significant

Generally, fewer edges unique to any single network were observed, particularly when considering edges between high-centrality symptoms of sadness, lassitude, and inability-to-feel, which were stable across all networks. Edges connecting to pessimistic thoughts were shared in TRD and non-responders. In all three networks, the strongest edges as well as nodes were not significantly stronger than each other, but only compared to the low-ranking edges and nodes (Supplementary Figure 5).

Networks with 27 edges (14 stable) and 20 edges (10 stable) were estimated in TRD and non-response. The strongest edges in TRD linked pessimistic thoughts to sadness and lassitude, while the strongest edge in non-response linked concentration difficulties to inability-to-feel. A unique edge was observed in non-responders, linking reduced-appetite to suicidal thoughts. In both TRD and non-responders, sadness ranked before lassitude, inability-to-feel, and pessimistic thoughts, followed by concentration difficulties in non-responders and tension in TRD. When considering only stable edges, relatively higher node strength was observed for lassitude and pessimism in TRD.

In patients with response, 20 edges (10 stable) were estimated. The strongest edge was estimated between sadness and pessimistic thoughts and a unique edge was observed between reduced-appetite and inability-to-feel. The highest node strength was observed in sadness, inability-to-feel, lassitude, concentration difficulties, and pessimism, irrespective of considering all or only stable edges.

### Longitudinal changes in network structure

Across treatment, both in responders and in TRD network strength declined (TRD: *S* = 1.17, *p* < 0.001; response: *S* = 1.16, *p* = <0.001) and structure changed (TRD: *M* = 0.2, *p* < 0.001; response: *M* = 0.26, *p* = 0.026). In contrast, in non-responders, no change in global strength or invariance was observed. Edges unique to specific phenotypes pretreatment either lost significance or were shared between networks posttreatment (Figure 2B).

Changes in edge weights for each treatment outcome are displayed in Supplementary Figure 6. In TRD, biggest reductions and loss of stability were observed for edges linking reduced-appetite and reduced-sleep, concentration difficulties and inability-to-feel, and pessimistic thoughts and suicidal thoughts. In contrast, the link from tension to pessimistic thoughts increased and reached stability posttreatment. Among edges that were stable both pre- and posttreatment, the link from tension to concentration difficulties was reduced significantly (Δ = 0.14, *p* = 0.01), while small increases were observed between pessimistic thoughts and sadness (Δ = 0.02, *p* > 0.05) and lassitude (Δ = 0.04, *p* > 0.05).

In responders, biggest reductions in edge weights were observed between reduced appetite and tension, between reduced appetite and concentration difficulties, between reduced appetite and suicidal-thoughts, between suicidal-thoughts and pessimistic-thoughts, between suicidal-thoughts and sadness, between lassitude and reduced-sleep, and between lassitude and pessimistic-thoughts. Edges increased between sadness and pessimistic-thoughts as well as between tension and both sadness and concentration difficulties, reaching stability only post-treatment.

## Discussion

We identified shared and outcome-specific changes in symptom connectivity when applying psychometric network modeling to a naturalistic cohort of 1,385 patients with MDD and treatment-response, non-response, and TRD.

Global network strength was not significantly different between groups pretreatment, albeit the highest scores were observed in TRD followed by non-response and lastly response. On one hand, higher symptom connectivity was associated with unfavorable outcomes such as higher risk for MDD onset [32], symptom persistence [20], treatment non-response [33, 34], or relapse following successful psychotherapy [35]. Contrary to prior suggestions of differences between outcome groups being driven by higher variance of mean symptom scores rather than phenotypic qualities [34], here lowest variance of average symptom load but highest global strength was observed in TRD. However, results must be interpreted cautiously as we cannot rule out that retrospective assessment of pretreatment symptoms introduced a bias to severity ratings.

On the other hand, increasing network strength throughout treatment was reported [33, 36, 37], likely due to symptoms decreasing simultaneously following successful treatment. However, some studies suggested persistence of network strength [38], depending on specific symptom associations [39]. Here, global strength was reduced across all groups following at least 4 weeks of antidepressant treatment. Thereby, posttreatment networks became more similar across groups, indicated by fewer edges being unique to a specific outcome and converging rankings regarding symptom strength. Interestingly, significant reductions in strength were found both in TRD and response but not in non-response. From a clinical perspective, the non-response network resembles an earlier stage of treatment compared to TRD patients who were treated for ≥4 additional weeks. Our results are thus suggestive of decreased global network strength relative to severity reduction and treatment duration. Consequently, some aspects of the TRD phenotype may be produced by partial response to sequential antidepressant trials that cure more easily treatable symptoms while those more resistant remain. Particularly in the case of unfavorable outcomes, connections between sadness, lassitude, inability-to-feel, and pessimistic thoughts densified posttreatment. While dense connections could theoretically also indicate a shared decrease of these four symptoms, here we observed high rates of persistence. Lassitude was frequent and severe specifically in the cohort of TRD patients, both pretreatment (83% with MADRS item score > 3, compared to 64–67% in other phenotypes) and posttreatment (90.7% with MADRS item score ≥ 3). Similarly, across all outcome phenotypes but particularly in TRD, high symptom loads of sadness were observed pretreatment (94% with MADRS item >3 in TRD, 78–82% in other phenotypes) and sadness showed higher rates of persistence than any other symptom (96.3% with MADRS item score ≥ 3 in TRD). The increased connectivity between these core symptoms despite the reduction of overall symptom load therefore seems to indicate resistance, and it may have higher clinical relevance than global changes in network strength.

Previous research on the factor structure of MADRS suggested one to four factors, but more recent analyses in large samples of severely depressed patients with MDD agreed on a four-factor model, including factors sadness, neurovegetative symptoms, detachment, and negative thoughts [40, 41]. This model presented with configural and metric invariance over antidepressant treatment, indicating that items maintained the direction and magnitude of their factor loadings. However, these results were based on samples with higher rates of treatment response and TRD has thus far not been investigated in this context. Reflecting on the current results, longitudinal factor patterns may differ between treatment outcome groups as patients with TRD appear to maintain higher symptoms related to factors “sadness” (sadness), “detachment” (lassitude and inability-to-feel), and “negative thoughts” (pessimistic thoughts) compared to other symptoms. These symptoms partly align with the proposed anhedonia factor of the MADRS, which also includes concentration difficulties while omitting pessimistic thoughts [42].

Regarding individual symptom scores, sadness, lassitude, pessimistic thoughts, and inability-to-feel were the most densely connected symptoms. While outcome-specific rankings of node strength were observed pretreatment, posttreatment these four symptoms showed the highest connectivity across all outcomes. Our results thus agree with a recent review on MDD symptom networks that identified fatigue as the top-ranked symptom regarding centrality strength [43]. Fatigue is a symptom describing reduced energy and exhaustion, thus corresponding well with lassitude assessed by the MADRS as in our study. Fatigue is also a symptom known for high persistence throughout treatment and as a residual symptom associated with the risk of relapse [44]. Strong connections linked lassitude to sadness and concentration difficulties across all outcomes, while a strong connection to pessimistic thoughts was found exclusively in TRD and particularly posttreatment. Fatigue was previously suggested to be part of somatic symptoms of depression, bridging them to emotional symptoms via anhedonia [43]. In line with this hypothesis, strong links were identified with inability-to-feel, which can be regarded as a representation of anhedonia, but also directly to sadness as well as to concentration difficulties. Anhedonia symptoms placing centrally all outcomes may also suggest that this subgroup of patients could benefit from combination treatments targeting anhedonia to possibly reduce both TRD risk and residual symptoms in responders [45].

Sadness, the other symptom consistently leading centrality rankings in MDD [38, 43], was the most strongly connected symptom posttreatment, agreeing with previous findings in the STAR*D sample [21]. Surprisingly, sadness placed below decreased-appetite and suicidal thoughts in centrality rankings of the pretreatment symptom network solely in the responder group. Both appetite changes and suicidality are typically placed among the least strongly connected symptoms in network analyses in MDD [43], which is also in line with our previous finding of HAM-D symptom reduced-appetite clustering only with reduced-weight but no other emotional or somatic symptoms in two naturalistic cohorts of depressed patients [14]. However, decreased appetite can be easily targeted by treatment, and response in this symptom domain was more easily predictable by machine learning classification models than other symptoms such as emotional or anxious symptoms [14, 46]. On a more speculative note, acute suicidality may trigger more targeted treatments such as ketamine, lithium augmentation, or electroconvulsive therapy, which were prescribed to some patients as part of the naturalistic treatment in the present patient cohort [47–49]. Consequently, a relatively lower strength of core symptoms such as sadness, lassitude, and inability-to-feel compared to other symptoms that could either be more directly ameliorated by treatment or guide more invasive treatment options may be indicative of higher chances for treatment response. However, following this hypothesis, also reduced-sleep could be expected to show higher centrality in treatment response, as it can be specifically targeted by sedating antidepressants and antipsychotics, but the opposite association was reported by previous research [34].

In summary, while previous discussions about the indications of centrality measures generally argued in favor of more central symptoms being more relevant to treatment [50], mechanisms behind this assumption may be less generalizable and dependent on specific symptom connections. On one hand, a symptom that is more densely connected to other symptoms can be a prime treatment target as lowering the symptom load may also reduce concomitant symptoms. However, more isolated symptoms with high perseverance throughout treatment may be particularly difficult to cure, as synergistic effects from neighboring symptoms are missing. The mechanisms involved may be dependent on the specific symptom edges present in an individual patient. As an example, a link between suicidality and worthlessness, the latter being here represented by pessimistic thoughts, was commonly observed in network analyses targeting MDD and also consistently observed here. However, this link was weaker in TRD compared to other outcome groups and lost significance in the TRD group after treatment. Whether the presence and strength of this connection has direct implications for treatment of either of the two symptoms remains to be resolved.

While we followed published recommendations regarding network generation and testing for stability and accuracy and took into consideration potential pitfalls such as Berkson’s bias, we cannot rule out spurious findings as the generalizability of the network results could not yet be validated in an independent sample. While a methodological framework for applying network structures to novel datasets has been published [23], to our knowledge there are no publicly available samples specifically addressing TRD. Further, limitations specific to the TRD-III data set must be considered. Results are based on a naturalistic sample of highly heterogeneous patients with a broad range of interventions applied in different treatment centers across Europe. While all interviews for MINI and MADRS were performed by specialist medical staff with training, inter-rater reliability was not tested and differences in ratings may have influenced the results. This concern is somewhat relieved by our prior studies based on the TRD-III dataset that showed consistent performance of models based on clinical characteristics when a cross-center validation design was applied [51]. Most importantly, medical documentation of baseline symptoms was not routinely available and the retrospective assessment of pretreatment MADRS scores relied on adequate recollection of the participating patients. While prior studies have shown good reproducibility regarding retrospective assessment of symptom types [52], recollection of symptom severity may be biased by the current disease state. On one hand, patients achieving response may have better insight and find it easier to identify past symptoms such as reduced-appetite. On the other hand, unsuccessful treatment outcomes and persistent symptom load may lead to overestimation of initial severity. However, mean baseline symptom scores were not statistically significant between groups, and also results regarding group differences in baseline networks are in line with clinical considerations and previous research [43]. Nevertheless, retrospective symptom assessment certainly introduces qualitative differences in networks estimated for pre- and posttreatment that cannot be accounted for by statistical methods. Particularly these results must therefore be regarded preliminary and need further validation by longitudinal study designs.

In synopsis, we observed differences in network structure between patients with MDD and treatment response, non-response, and TRD for symptoms reported both pretreatment and posttreatment. Symptom connections including pessimistic thoughts were particularly pronounced in TRD, while symptoms of reduced-appetite and suicidal thoughts were more densely connected in the response group. Posttreatment, global strength was higher in patients with TRD and in non-responders compared to responders, but network structures became more similar and significant edges were mostly shared between outcome groups. Despite overall decreases in network strength following treatment, specific connections between core symptoms of sadness, lassitude, inability-to-feel, and pessimistic thoughts increased. Particularly in TRD, these symptoms showed high rates of persistence after treatment and patients may benefit from targeted treatment approaches.

## Supporting information

10.1192/j.eurpsy.2025.2454.sm001 Kautzky et al. supplementary material Kautzky et al. supplementary material

## Data Availability

The data that support the findings of this study contain sensitive information that could compromise the privacy of research participants and are therefore are not publicly available. The code used to generate the results as well as a randomly generated sample similar in design to the original data is available as Supplemental Material. The original data that were used to support the findings of this study are available from the corresponding author S.K. upon reasonable request.

## Supplementary material

The supplementary material for this article can be found at http://doi.org/10.1192/j.eurpsy.2025.2454.
