# Supplementary material for: Symptom networks in major depressive disorder and treatment response: special focus on TRD
Source: Eur Psychiatry. 2025 May 27;68(1):e61. doi: 10.1192/j.eurpsy.2025.2454 (PMC12260719; doi:10.1192/j.eurpsy.2025.2454)
Supplement: Kautzky et al. supplementary material [file S092493382502454Xsup001.zip › networksTRD_supplements 161224.docx]

**Network Stability and Edge Weight Accuracy**

Following published guidelines for using the “bootnet” function of the synonymous package [1], *case-dropping subset bootstrap* with 1000 iterations was performed with subsamples covering 0.05 to 0.75% of the full data set. was performed over. Applying this method yields correlations of network statistics with the original sample with 95% confidence intervals (CI). The coefficient termed *correlation-stability* (CS) coefficient was computed, giving the proportion of cases that can be dropped while maintaining a correlation ≥ 0.7 between edge weights within 95% of the bootstrapped samples. Based on recommendations [1], we defined CS thresholds > 0.25 and > 0.5 indicating acceptable and good stability respectively. In other words, good stability (CS > 0.5) requires that at least half of the sample could be dropped fulfilling this condition.

Further using the “bootnet” function and published guidelines[1], non-parametric bootstrapping with 1000 iterations was performed to estimate the distribution of confidence intervals (CI) for each individual edge weight. While not recommended for testing significance of edge weights, this method assesses the accuracy of network estimates. Here, all edges with non-zero CI were considered stable for further comparison between networks. Finally, comparisons of edge weights within each network were done to conclude if one edge weight was significantly stronger than another present in the same network. Thereby, we computed CI of the differences between each pair of edge weights across bootstrapping. In case of CI not including 0, significant difference in strength was assumed. Similar procedures were used to assess stability and accuracy for node strength. Based on considerations on statistical feasibility [1], no correction for multiple testing was applied.

**Assessment of Berkson´s bias**

Simulation studies supported a critical role of Berkson´s bias in network modelling [2]. Using a threshold of a depression severity rating scale to define groups for network modelling of depressive symptoms may introduce spurious negative edges in these groups that are not present in the complete sample. To assess stability of symptom correlations between the complete sample and the three groups of treatment response, non-response and treatment-resistant depression (TRD), we computed correlation coefficients between the two exemplary symptoms of sadness and lassitude. Assessment of Berkson´s bias revealed consistent positive correlations between sadness and lassitude in the complete sample as well as all three groups, respectively for symptoms reported pre- and post-treatment (Supplementary Figure 7). Thus, we concluded that the group definition did not introduce spurious negative correlations attributable to Berkson´s bias.

|  | **Response (n=326)** | **TRD (n=570)** | **Non-Response (n=489)** | **Overall (n=1385)** |
| --- | --- | --- | --- | --- |
| **Sadness** |  |  |  |  |
| Item Score 1-3 | 72 (22.1%) | 36 (6.3%) | 88 (18.0%) | 196 (14.2%) |
| Item Score > 3 | 254 (77.9%) | 534 (93.7%) | 401 (82.0%) | 1189 (85.8%) |
| Persistent | 23 (9.1%) | 517 (96.8%) | 378 (94.3%) | 918 (77.2%) |
| **Tension** |  |  |  |  |
| Item Score 1-3 | 162 (49.7%) | 268 (47.0%) | 224 (45.8%) | 654 (47.2%) |
| Item Score > 3 | 164 (50.3%) | 302 (53.0%) | 265 (54.2%) | 731 (52.8%) |
| Persistent | 19 (11.5%) | 264 (87.4%) | 213 (80.4%) | 496 (67.9%) |
| **Reduced-Sleep** |  |  |  |  |
| Item Score 1-3 | 133 (40.8%) | 180 (31.6%) | 185 (37.8%) | 498 (36.0%) |
| Item Score > 3 | 193 (59.2%) | 390 (68.4%) | 304 (62.2%) | 887 (64.0%) |
| Persistent | 11 (5.7%) | 335 (85.9%) | 246 (80.9%) | 592 (66.7%) |
| **Reduced-Appetite** |  |  |  |  |
| Item Score 1-3 | 211 (64.7%) | 397 (69.6%) | 383 (78.3%) | 991 (71.6%) |
| Item Score > 3 | 115 (35.3%) | 173 (30.4%) | 106 (21.7%) | 394 (28.4%) |
| Persistent | 6 (5.2%%) | 114 (65.9%) | 66 (62.3%) | 186 (47.2%) |
| **Concentration-Difficulties** |  |  |  |  |
| Item Score 1-3 | 177 (54.3%) | 248 (43.5%) | 227 (46.4%) | 652 (47.1%) |
| Item Score > 3 | 149 (45.7%) | 322 (56.5%) | 262 (53.6%) | 733 (52.9%) |
| Persistent | 20 (13.4%) | 280 (87.0%) | 226 (86.3%) | 526 (71.8%) |
| **Lassitude** |  |  |  |  |
| Item Score 1-3 | 117 (35.9%) | 100 (17.5%) | 162 (33.1%) | 379 (27.4%) |
| Item Score > 3 | 209 (64.1%) | 470 (82.5%) | 327 (66.9%) | 1006 (72.6%) |
| Persistent | 11 (5.3%) | 427 (90.9%) | 282 (86.2%) | 720 (71.6%) |
| **Inability-to-Feel** |  |  |  |  |
| Item Score 1-3 | 168 (51.5%) | 186 (32.6%) | 225 (46.0%) | 579 (41.8%) |
| Item Score > 3 | 158 (48.5%) | 384 (67.4%) | 264 (54.0%) | 806 (58.2%) |
| Persistent | 4 (2.5%) | 346 (90.1%) | 214 (81.1%) | 564 (70.0%) |
| **Pessimistic-Thoughts** |  |  |  |  |
| Item Score 1-3 | 176 (54.0%) | 199 (34.9%) | 253 (51.7%) | 628 (45.3%) |
| Item Score > 3 | 150 (46.0%) | 371 (65.1%) | 236 (48.3%) | 757 (54.7%) |
| Persistent | 14 (9.3%) | 344 (92.7%) | 196 (83.1%) | 554 (73.2%) |
| **Suicidal-Thoughts** |  |  |  |  |
| Item Score 1-3 | 260 (79.8%) | 448 (78.6%) | 392 (80.2%) | 1100 (79.4%) |
| Item Score > 3 | 66 (20.2%) | 122 (21.4%) | 97 (19.8%) | 285 (20.6%) |
| Persistent | 2 (3.0%) | 89 (73.0%) | 51 (52.6%) | 142 (49.8%) |

**Supplemental Table 1.** Mild to moderate (item score 1-3) and severe (item score > 3) symptoms before treatment initiation, respectively among patients with treatment response, non-response and TRD. In addition, the percentages of patients with persistent symptoms (item score post-treatment ≥ 3) among those with severe symptoms pre-treatment are provided.


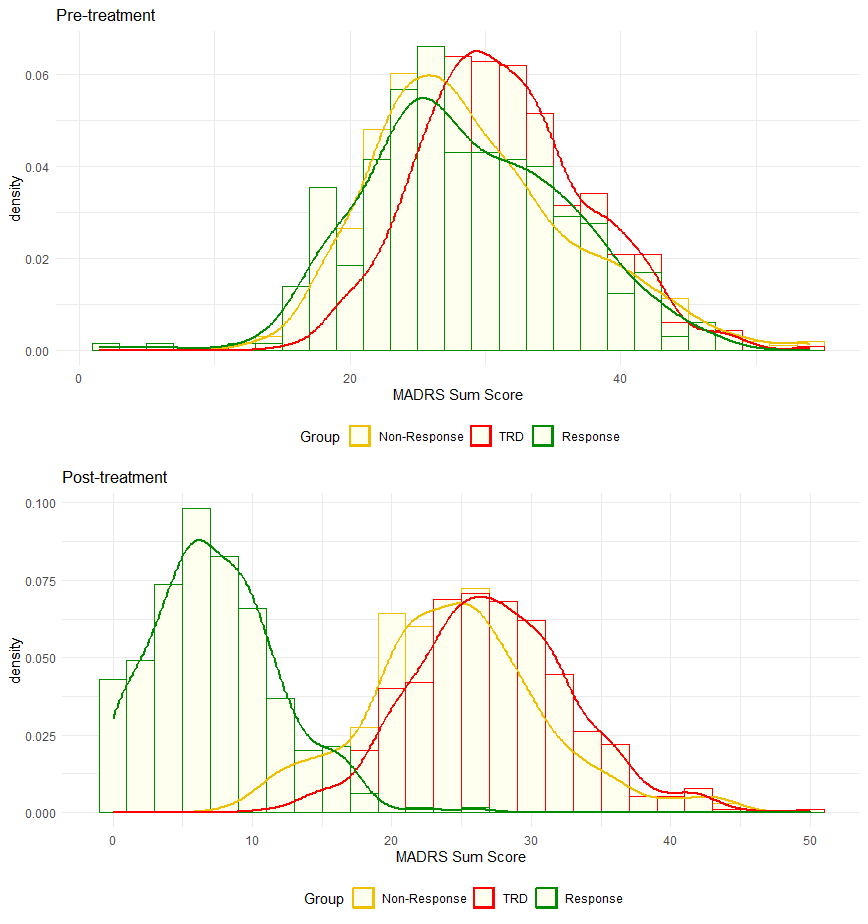


**Supplementary Figure 1.** Distribution of Montgomery-Åsberg depression rating scale (MADRS) sum scores among the three groups of treatment resistance (TRD), non-response and response. Pre-treatment, largely overlapping distributions were observed, while post-treatment a shift to the left and a skewed distribution was observed among patients with treatment response.


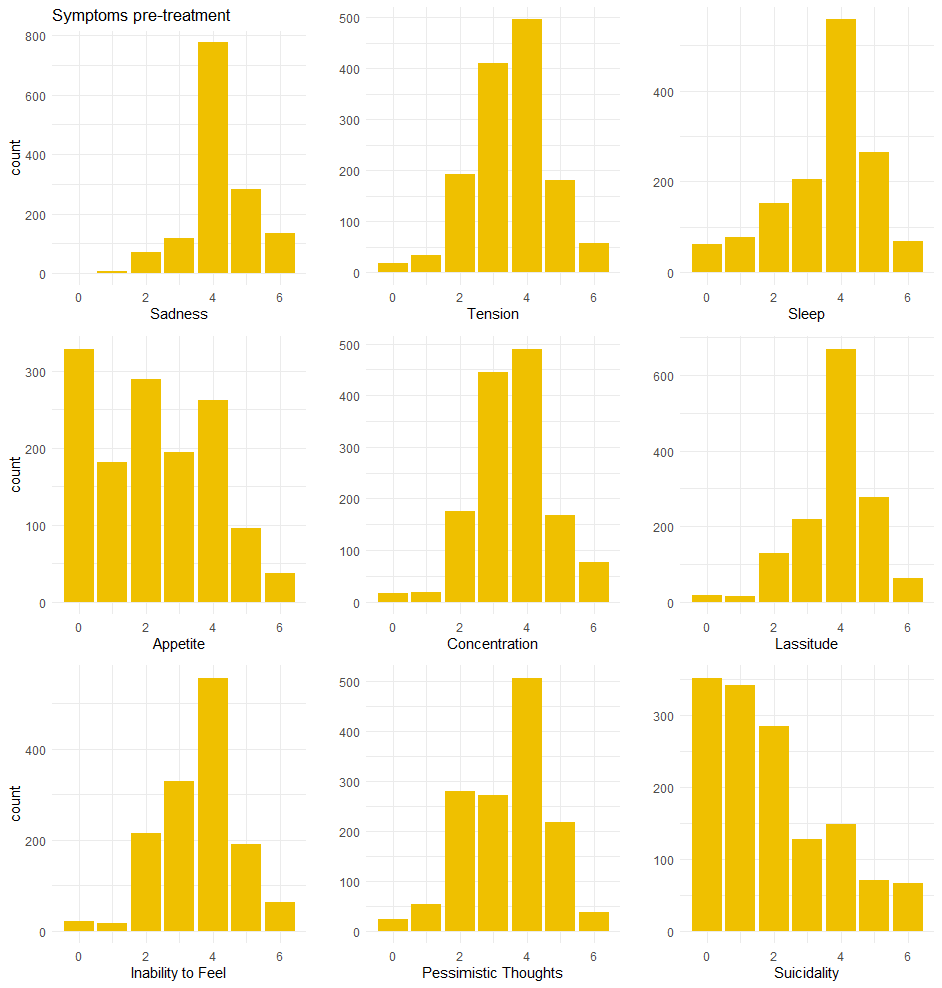


**Supplementary Figure 2.** Distribution of Montgomery-Åsberg depression rating scale (MADRS) items for symptoms reported pre-treatment.


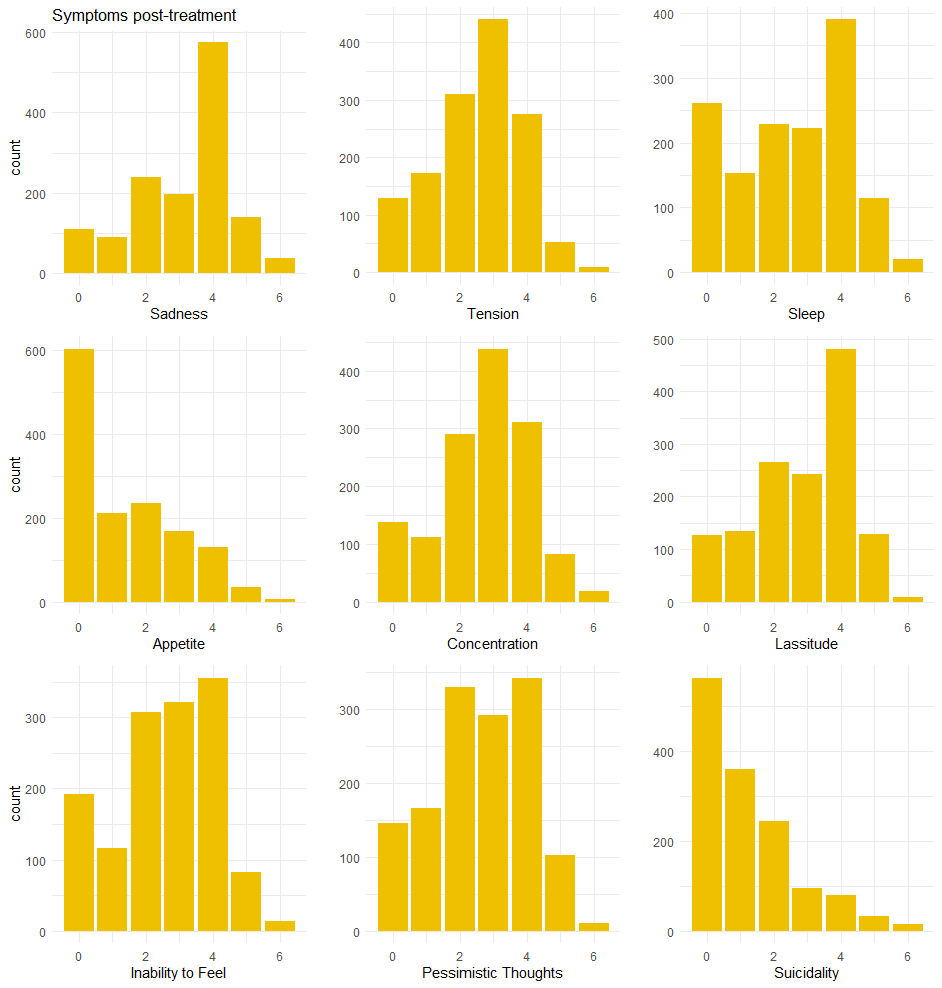


**Supplementary Figure 3.** Distribution of Montgomery-Åsberg depression rating scale (MADRS) items for symptoms reported post-treatment.


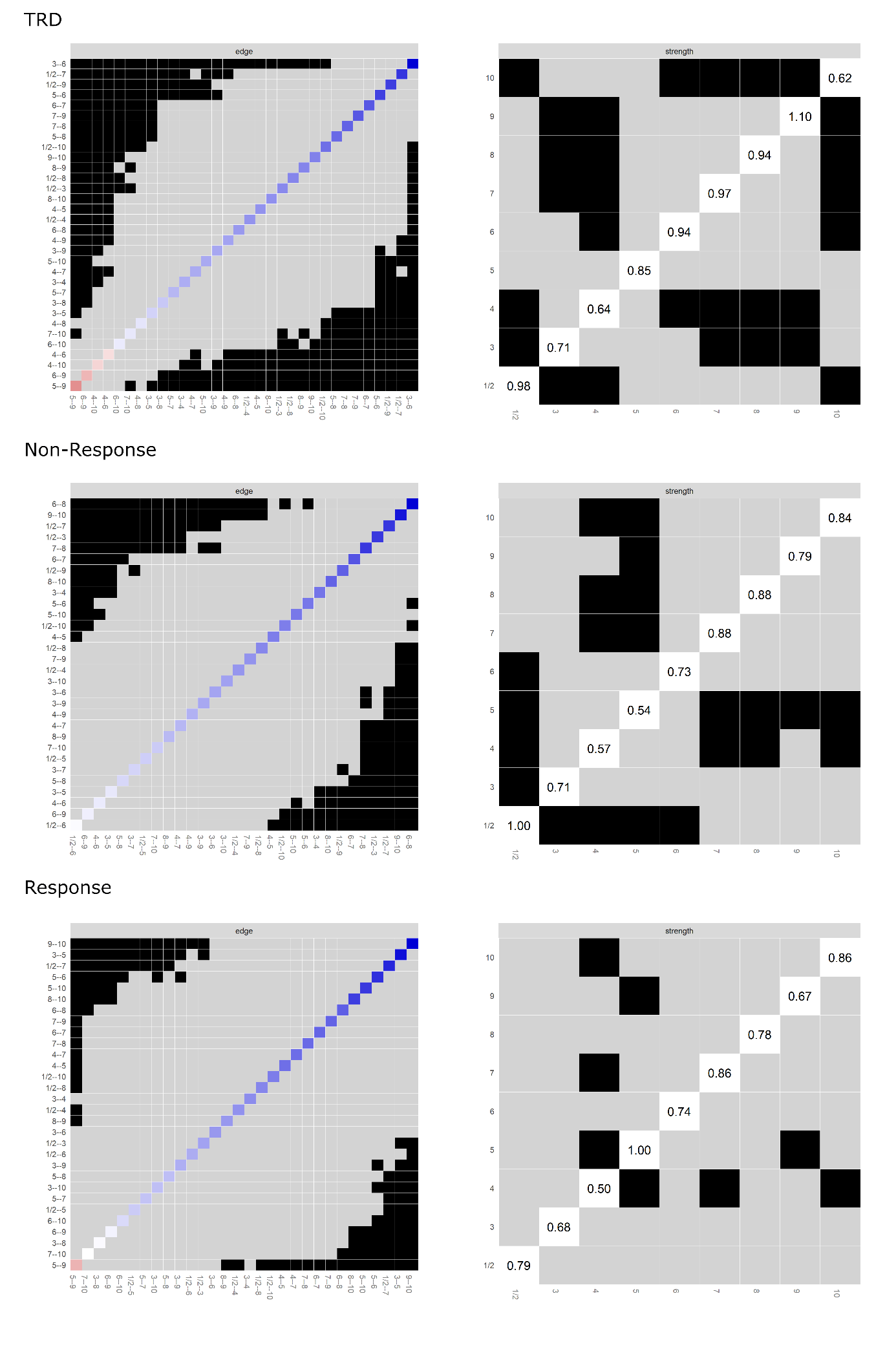


**Supplementary Figure 4.** Bootstrapping results for networks estimated for pre-treatment symptoms. Symptoms are named according to their respective MADRS item number. Items 1 and 2 were averaged as they both measure sadness. Respectively for each outcome group, differences between edges are shown on the left panel and differences between centrality index strength on the right panel. The difference in each pair of respectively edge weights and node strengths was computed across 1000 iterations of bootstrapping. Whenever confidence intervals did not include 0, the two edges or nodes were considered significantly different from each other. Black coloring indicates significantly different edge weights or node strength for the respective comparison.


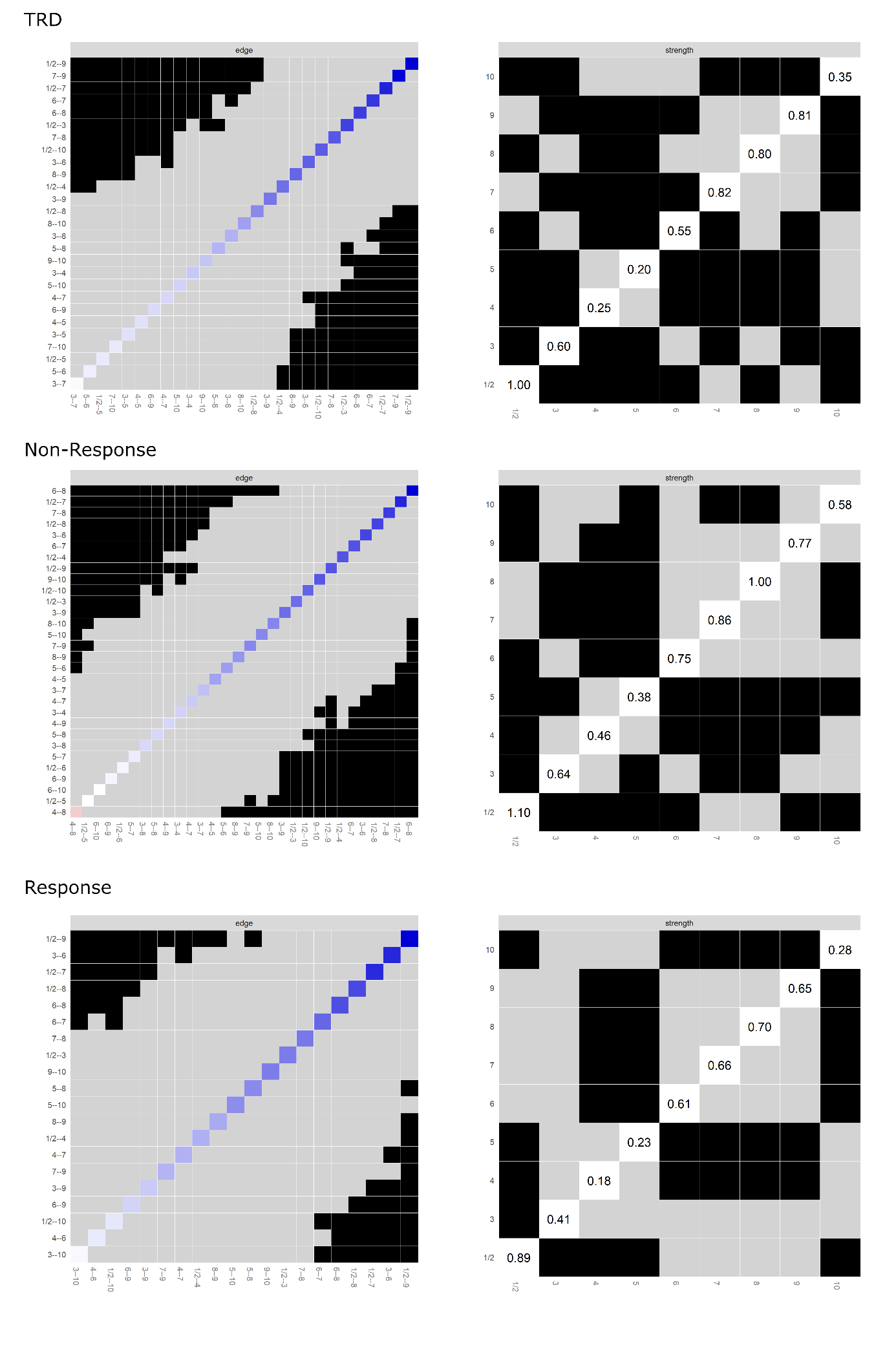


**Supplementary Figure 5.** Bootstrapping results for networks estimated for post-treatment symptoms. Symptoms are named according to their respective MADRS item number. Items 1 and 2 were averaged as they both measure sadness. Respectively for each outcome group, differences between edges are shown on the left panel and differences between centrality index strength on the right panel. The difference in each pair of respectively edge weights and node strengths was computed across 1000 iterations of bootstrapping. Whenever confidence intervals did not include 0, the two edges or nodes were considered significantly different from each other. Black coloring indicates significantly different edge weights or node strength for the respective comparison.


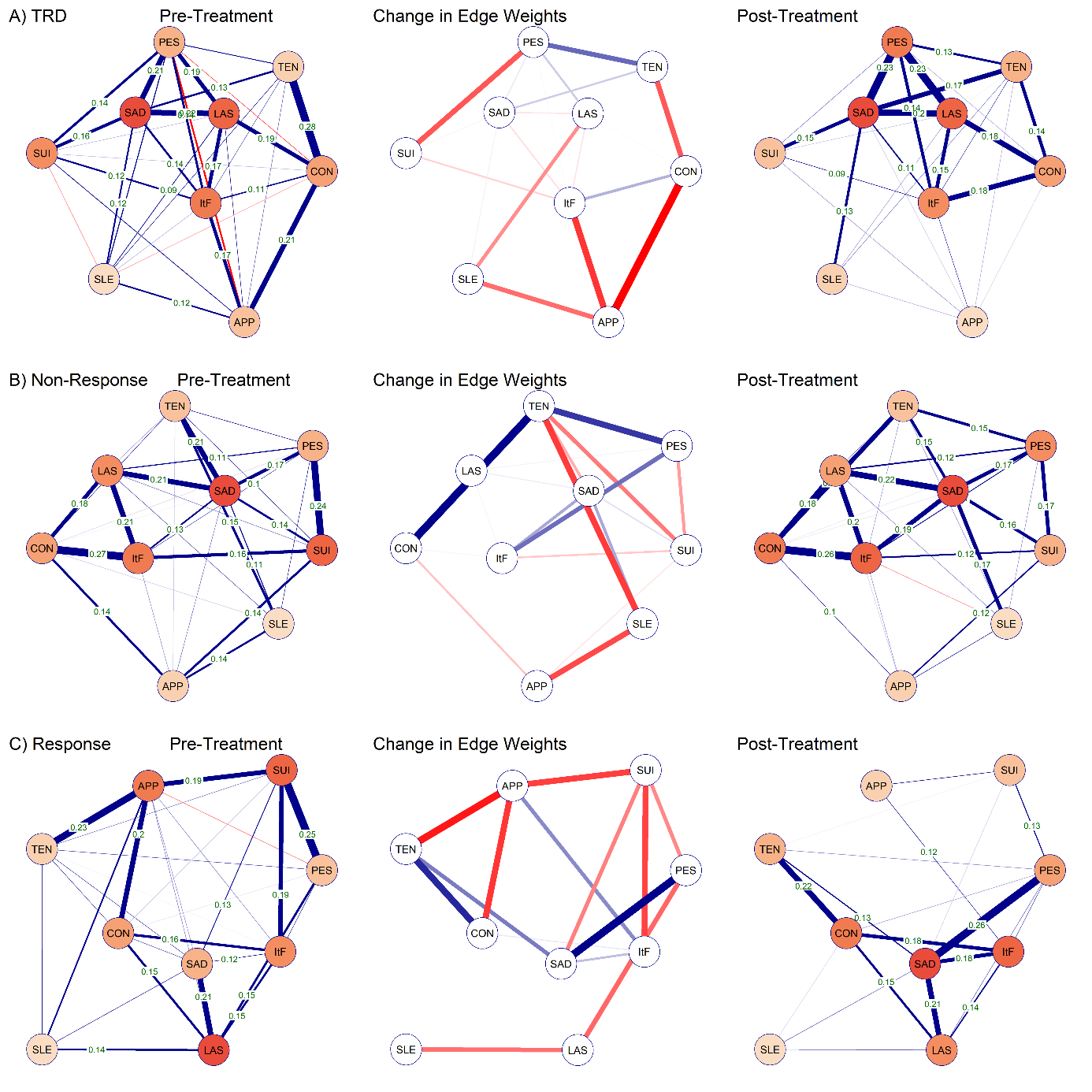


**Supplementary Figure 6.** Gaussian graphical models estimated respectively for symptoms before initiation of antidepressant treatment (left column) and after treatment outcome was determined (right column), A) for patients with TRD, B) non-response and C). Positive partial correlations, i.e., co-expression of severity of two symptoms, are portrayed in blue color while negative partial correlations, i.e., high load of one symptom occurring with low severity of another, are portrayed in red color. In the middle column, absolute differences in edge weights are portrayed respectively in red (reduced edge weights) and blue (increased edge weights). In all networks, edge weights are only displayed when stable according to bootstrapping. Nodes are colored dark to light by declining node strength, i.e., the sum of edge-weights connecting each node.

Abbreviations: APP = reduced-appetite, CON = concentration‑difficulties, ItF = inability-to-feel, LAS = lassitude, PES = pessimistic-thoughts, SAD = sadness, SLE = reduced-sleep, SUI = suicidal‑thoughts, TEN = tension, TRD = treatment resistant depression

**
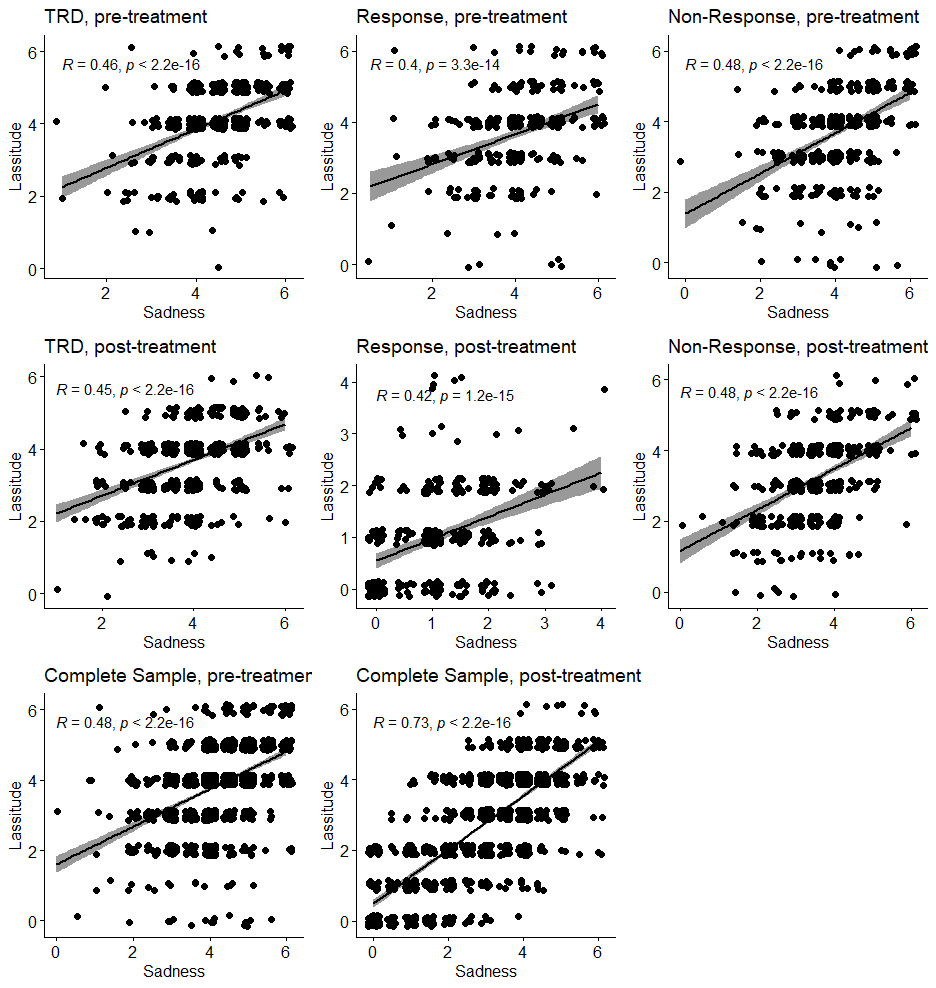
**

**Supplementary Figure 7.** Correlation between the Montgomery-Åsberg depression rating scale (MADRS) scores of two depressive symptoms sadness and lassitude, respectively among the complete sample of 1385 patients and among the three groups of treatment resistance (TRD), non-response and response. Correlations were computed respectively for symptoms reported for pre- and post-treatment. Symptoms were positively correlated across all samples, suggesting that Berkson´s bias was

**References**

[1] Epskamp S, Borsboom D, Fried EI. Estimating psychological networks and their accuracy: A tutorial paper. Behav Res Methods. 2018;50:195-212.

[2] de Ron J, Fried EI, Epskamp S. Psychological networks in clinical populations: investigating the consequences of Berkson's bias. Psychol Med. 2021;51:168-76.
